# Enhancement of the Physicochemical Properties of Brazilian Red Propolis Using Gelucire-Based Microencapsulation

**DOI:** 10.1021/acsomega.5c08627

**Published:** 2026-01-19

**Authors:** Jesudunni Aanuolu Akinola, Wedja Timóteo Vieira, Wanderley Pereira Oliveira

**Affiliations:** School of Pharmaceutical Sciences of Ribeirão Preto, University of São Paulo, Ribeirão Preto 14040-903, Brazil

## Abstract

Red propolis is renowned for its versatility as a natural product with numerous pharmacological activities. However, its pharmacological efficacy is constrained by its hydrophobic nature, leading to poor oral bioavailability and absorption. Encapsulation, particularly through spray-drying, offers a promising strategy to overcome this limitation by improving solubility and stability. This study evaluated the impact of Gelucire 50/13 (G), a surfactant known for enhancing the aqueous solubility and bioavailability of hydrophobic drugs, on the physicochemical properties of encapsulated red propolis extract (RPE). The extract was characterized to determine its solid content and total flavonoid content (TFC). Encapsulation of RPE in Gelucire was followed by spray-drying using three carriers: Arabic gum, octenyl succinic anhydride (OSA)-modified starch, and maltodextrin. For comparison, plain RPE was also spray-dried using the same carriers. The encapsulated formulations were characterized for their physicochemical properties, including water activity, moisture content, and solubility, as well as qualitative chemical integrity, assessed using HPLC analysis. The TFC of RPE was determined to be 13.11 mg of the quercetin equivalent. RPE-Gelucire formulations demonstrated improved spray-drying yields and superior physicochemical properties compared to plain RPE formulations. Additionally, HPLC analysis confirmed the preservation of the extract’s chemical profile postencapsulation. These findings highlight the potential of Gelucire-based spray-drying encapsulation with carriers to enhance the solubility, stability, and physicochemical properties of the Brazilian red propolis extract.

## Graphical abstract

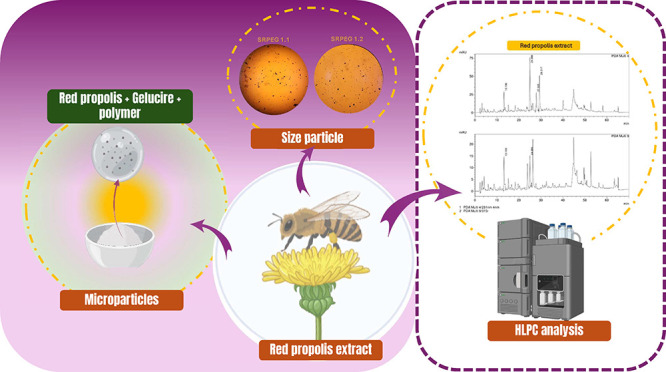

## 1 Introduction

Red propolis is a distinctive red-colored sticky resin produced by honeybees ( *Apis mellifera* ) through the mixing of plant exudates with beeswax, pollen, and some enzymes. The resin is primarily produced to protect the hive from intruders and to prevent contamination.^1,2^ The primary botanical origin of red propolis is *Dalbergia ecastyphyllum*, a leguminous shrub commonly found in mangrove ecosystems, particularly along the northeast coast of Brazil. The unique environmental conditions of this region contribute to the resin’s chemical profile, making it a complex mixture rich in phenolic compounds, flavonoids, and terpenes. These components are responsible for their diverse biological activities, including antimicrobial, antifungal, antiviral, cytotoxic, antioxidant, and immunostimulant properties.^2–4^ Formononetin, quercetin, Liquiritigenin, and biochanin A are characteristic marker compounds of red propolis, which have been linked to its therapeutic effects.^5^

Despite its versatility, red propolis’s hydrophobic nature limits its solubility, bioavailability, and absorption, thereby reducing its pharmacological efficacy.^6^ This drawback presents significant challenges for its use in pharmaceutical and nutraceutical applications. Enhancing its solubility and bioavailability could improve its effectiveness across various applications, including antimicrobial, antioxidant, immunostimulatory, and anticancer treatments. Recent formulation strategies, including spray-drying with carbohydrate carriers, spray-chilling, and polymeric nanoparticle systems, have attempted to improve dispersibility, stability, and controlled release of propolis extracts.^7,8^ However, these systems often involve multistep processing or rely on complex excipient combinations, leaving room for simpler and more efficient solubilizing carriers. Dispersion followed by encapsulation offers a streamlined and effective strategy to address these challenges while preserving the chemical integrity of the bioactive markers. Furthermore, several studies in the literature report the use of excipients, such as Gelucire 50/13.

Gelucire 50/13, an amphiphilic lipid excipient composed of PEG-esters, a small glyceride fraction, and free PEG, has been widely studied as a solubility-enhancing agent because, upon contact with aqueous media, it self-emulsifies to form fine dispersions that enhance the solubility and dissolution of poorly water-soluble compounds.^9^ It can self-emulsify in aqueous media, forming fine dispersions or microemulsions. Gelucire 50/13 is classified as Generally Recognized As Safe (GRAS) by both the European and the United States Pharmacopoeia and has been extensively used in pharmaceutical formulations to improve the solubility, dissolution, and bioavailability of hydrophobic compounds.^10,11^ In addition, its dual lipid–PEG structure enables both molecular dispersion of hydrophobic actives and protection of sensitive constituents from degradation. Several studies have demonstrated its versatility, including applications for meloxicam, cefuroxime axetil, and aceclofenac, which significantly enhanced dissolution and absorption.^11,12^ Gelucire 50/13 has also been successfully applied to natural antioxidants such as curcumin and ferulic acid, demonstrating its ability to enhance solubility, preserve bioactivity, and improve systemic exposure.^13,14^ Unlike other amphiphilic carriers such as poloxamers or PEG-based surfactants, Gelucire 50/13 offers a unique combination of solubilization, lipidic protection, and self-emulsifying behavior without requiring cosolvents or multiple surfactants.^15^ These properties make it particularly suited for complex natural extracts. These precedents support its suitability for complex, phytochemical-rich extracts such as Brazilian red propolis.

Encapsulation is a technique that encloses an active substance within a shell-forming material, allowing its controlled release under specific conditions, such as pH, water activity, temperature, or time.^16^ This technique is widely used to address the limitations of bioactive phytochemicals, which often suffer from low bioavailability, poor aqueous solubility, and stability. Encapsulation in biodegradable and biocompatible nano or microparticles has been shown to enhance these properties, improve absorption, and increase target specificity, ultimately increasing the pharmacological activity of bioactive compounds^16,17^

Spray drying is one of the most commonly used encapsulation techniques due to its efficiency in producing stable powders. The process involves atomizing a liquid formulation into a stream of hot gas, which facilitates rapid water evaporation while maintaining low internal particle temperatures. These characteristics make spray drying particularly suitable for heat-sensitive compounds like red propolis. Prior to spray drying, the liquid formulation containing the wall material and the bioactive compound is homogenized to ensure uniform encapsulation. Common encapsulation materials include gum arabic, chemically modified starch, maltodextrin, soy protein, whey protein, and gelatin.^18,19^ In this study, gum arabic, octenyl succinic anhydride (OSA)-modified starch, and maltodextrin were selected due to their emulsifying properties, solubility, and cost-effectiveness.

When combined with spray drying, Gelucire 50/13 is expected to act synergistically with wall materials such as gum arabic, OSA-modified starch, and maltodextrin, collectively enhancing encapsulation efficiency and stability. This approach aims to improve the physicochemical properties of the encapsulated extract while preserving its key bioactive markers. Brazilian red propolis is a hydrophobic ethanolic extract rich in flavonoids and isoflavonoids, compounds known for poor water solubility and sensitivity to oxidation. Gelucire 50/13 provides an optimal encapsulation matrix by promoting solubilization through self-emulsification, stabilizing bioactives during spray drying, and supporting the development of powders with enhanced reconstitution and bioavailability. These properties justify its selection for encapsulating Brazilian red propolis in oral and nutraceutical formulations. To the best of our knowledge, this is the first study to demonstrate the use of Gelucire 50/13 as a lipid-based delivery system for Brazilian red propolis. This excipient serves as both a solubilizing and encapsulating matrix, offering a novel strategy to address the inherent lipophilicity of the extract’s bioactive constituents and enhance their potential for oral bioavailability.

This study aims to encapsulate red propolis extract using Gelucire 50/13 and spray drying, evaluate the physicochemical properties of the encapsulated extract, assess the retention of key bioactive compounds, such as formononetin and liquiritigenin, and compare the performance of gum arabic, OSA-modified starch, and maltodextrin as wall materials. Given red propolis’s hydrophobic nature, this optimized formulation is proposed to improve its applicability and potential for oral use in pharmaceutical, nutraceutical, and cosmetic products, as well as in functional foods and supplements, veterinary medicine, and the food industry.

## 2 Materials and Methods

### 2.1 Materials

Red Propolis Extract (RPE) was obtained from Rubee Apis Ltd. (Barra de Santo Antônio, Alagoas, Northeast Brazil). The RPE was produced by maceration using a proprietary industrial method; therefore, detailed extraction parameters are not disclosed. However, the extract underwent physicochemical characterization (Section 2.2.1) to ensure standardization and quality consistency for this study. The wall encapsulation and drying carrier materials used were Gelucire 50/13, Arabic gum, Maltodextrin 1920, and OSA modified starch (Capsul). For the physicochemical analyses and chromatographic assays, Quercetin, Ethanol (Labsynth), Aluminum chloride, and HPLC-grade reagents (Acetonitrile, formic acid, and Methanol) were used, along with Milli-Q water.

#### 2.1.1 Physicochemical Characterization of the Red Propolis Extract

##### 2.1.1.1 Determination of the Solids Content

2.0 g of Red Propolis extract was placed on a foil plate, and the solid content was determined using a moisture analyzer (SARTORIUS AG, Germany, MA 35M), in which approximately 2 g of the extract was evaporated to a constant weight at 105 °C. The test was conducted in triplicate, and the solid content was calculated using eq 1.

$$C_{s}=\frac{m}{P}\times 100$$ 1

where C_s_ = solid content (% m/m), m = mass of dried residue (g), and P = mass of sample (g).

##### 2.1.1.2 Determination of the Total Flavonoid Content (TFC)

The TFC was determined by UV–vis spectrophotometry using a HP 8453 spectrophotometer with the HP Chem-Station software (Agilent Life Sciences and Chemical Analysis, Santa Clara, CA, USA). This method is based on the absorbance displacement caused by the reaction with a 0.5% (w/v) AlCl_3_ solution.^20,21^ 25 mg of dry solid mass of propolis was weighed and dissolved in 50 mL of 40% ethanol, then stirred for 15 min. The extractive solution was filtered. The first 20 mL was discarded, and the remaining 30 mL was collected. After this, 4 mL of the sample was placed in a 10 mL volumetric flask, and 800 μL of aluminum chloride was added, and the solution was made up to the mark with 40% ethanol. The absorbance was measured at 425 nm, after 30 min of reaction. An analytic curve of quercetin was built, and TFC was expressed as mg of quercetin/g of extract (dried base). All samples were analyzed in triplicate.

#### 2.1.2 Obtaining and Characterizing Gelucire 50/13 Dispersion Loaded with the RPE

Red propolis extract (RPE) was encapsulated in Gelucire 50/13 using a dropwise dispersion method. Initially, 80 mL of RPE was added dropwise to 400 g of Gelucire 50/13 (5% w/w) using a peristaltic pump at a flow rate of 0.5 mL/min. The mixture was stirred at high speed on a magnetic stirrer for 1 h to ensure preliminary dispersion.

Following magnetic stirring, the formulation was transferred to an Ultraturrax homogenizer (T-18 IKA Works, Inc., Wilmington, NC, USA) and homogenized at 20,000 rpm for 5 min to enhance dispersion. The homogenized mixture was then subjected to ultrasonication using a VCX-750 SONICS Vibracell ultrasonic processor (New Town, USA) at 45% amplitude for 5 min. This step was designed to reduce particle size and improve encapsulation efficiency, yielding the final RPE-Gelucire formulation (RPEG).

Samples were collected at three stages of the process (postmagnetic stirring, posthomogenization, and postultrasonication) and stored at room temperature for 24 h prior to analysis of particle size, polydispersity index (PDI), and zeta potential (ζ-potential). Particle size distribution and PDI were determined using Dynamic Light Scattering (DLS), while ζ-potential was measured by microelectrophoresis using a Zetasizer Nano-ZS90 (Malvern, UK). Samples were diluted 1:500 (v/v) in Milli-Q water and stirred for 30 min before measurement. All analyses were performed in triplicate at 25 °C, and results were expressed as mean ± standard deviation.^22^

#### 2.1.3 Spray Drying/Microencapsulation of RPE and RPEG

Spray drying was employed to microencapsulate red propolis extract (RPE), and RPE encapsulated in Gelucire (RPEG) using arabic gum (GA), maltodextrin (MDX), and octenyl succinic acid-modified starch (S) as carriers, following the method of Zhang et al.^23^ with modifications. The carrier-to-active ingredient ratios in the formulations were systematically varied to assess their impact on encapsulation efficiency and physicochemical properties (Table 1). Each mixture was homogenized using an Ultraturrax (T-18 IKA Works, Inc., Wilmington, NC, USA) at 5,000 rpm for 15 min to ensure uniform dispersion, followed by magnetic stirring at 1,000 rpm for 30 min.

**Table 1:** Composition of the Spray-Dried Formulations and Their Respective Carrier-to-Active Ingredient Ratios

Formulation	Ratio
GA/RPEG	1:1
GA/RPEG	1:2
GA/RPE	1:1
GA/RPE	1:2
S/RPEG	1:1
S/RPEG	1:2
S/RPE	1:1
S/RPE	1:2
GA/MDX/RPE	1:2
MDX/RPEG	1:3

The feed suspensions were processed using a Lab-Plant SD-05 spray dryer (Lab-Plant, UK) equipped with a 250 mm drying chamber diameter, under optimized conditions: inlet temperature of 100 °C (selected to minimize thermal degradation of heat-sensitive bioactives), atomizing flow rate of 17 L/min at an air pressure of 2 kgf/cm^2^, nozzle diameter of 1.0 mm, and a feed flow rate of 4 g/min. The spray-dried powders were retrieved from the collection chamber and immediately stored in sealed, water-resistant aluminum foil pouches at 4 °C for further analysis. The yield of the spray-drying process was calculated as the percentage ratio of the weight of spray-dried powders to the total solids in the feed suspension, as described in USP guidelines.^24^

#### 2.1.4 Physicochemical Analysis of Spray-Dried Powders

The physicochemical properties of the spray-dried powders were analyzed to evaluate their suitability for pharmaceutical and nutraceutical applications. The analyses included moisture content, water activity, particle size distribution, powder flow properties, aqueous solubility, and HPLC fingerprint. Each measurement was conducted in triplicate unless otherwise specified, and results were expressed as mean ± standard deviation.

##### 2.1.4.1 Moisture Content

Moisture content was determined using Karl Fischer titration, following standard procedures with a Karl Fischer 870 Titrino Plus (Methrom, Switzerland). A 10 mg sample of powder was dissolved in methanol before titration. Duplicate measurements were performed, and the results were expressed as the mean ± standard deviation.

##### 2.1.4.2 Water Activity (A_w_)

The water activity of the spray-dried powders was measured at 25 °C using an AquaLab 4TEV instrument (Decagon Devices Inc., Pullman, WA) equipped with a dew point sensor. Approximately 1 g of powder was placed in the sample holder, and measurements were performed in triplicate; results were expressed as the mean and standard deviation.

##### 2.1.4.3 Particle Size Distribution

The particle size distribution of the spray-dried powders was determined using light microscopy, following the methods of Baldim et al. and Alamilla-Beltrán et al.,^22,25^ with modifications. Approximately 5 mg of powder was evenly dispersed onto a glass microscope slide without clumping. Images of the dispersed particles were captured using an optical microscope (Olympus BX60MIV, Tokyo, Japan) coupled to image analysis software (Image-Pro Plus 4.5, Media Cybernetics Inc., Bethesda, Rockville, MD, USA) to determine particle diameters. Measurements were performed at 50× magnification across multiple fields of view to ensure proper size representation. The D10, D50, D90, and SPAN values were calculated to assess the particle size distribution using the following eq 2.

$$SPAN=\frac{D90-D10}{D50}$$ 2

##### 2.1.4.4 Flow Properties of the Spray-Dried Powders

The flowability of the powders was determined by calculating the Hausner’s Ratio (HR) and Carr’s Index (CI) from the bulk and tapped densities. The bulk density was determined by gently placing a precisely weighed amount of the spray-dried powders (*m*_*0*_) into a 10 mL measuring cylinder; the volume occupied (*V*_*0*_) was noted, and the density (*d*_*0*_ *= m*_*0*_/*V*_*0*_) was calculated. The tapped density (*d*_*1250*_), which is powder density after tapping the cylinder 1250 times in accordance with USP guidelines,^24^ was measured using a Caleva Tapped Density Tester Type TDT (Frankfurt, Germany). The Hausner Ratio (HR) and Carr’s Index (CI) were determined using eqs 3 and 4, respectively.

$$HR=\frac{d1250}{d0}$$ 3

$$CI=\frac{d1250-d0}{d1250}\times 100$$ 4

##### 2.1.4.5 Aqueous Solubility of Powders

The solubility of the spray-dried powders was determined using a modified version of Milton et al.^26^ A 500 mg powder sample was added to 50 mL of distilled water in a beaker and stirred on a magnetic stirrer at 30 °C for 25 min. The resulting suspension was centrifuged at 7000 rpm for 10 min, and the supernatant was filtered through Whatman No. 1 filter paper. The filtrate was oven-dried at 105 °C for 4 h, and the percentage solubility was calculated as follows:

$$S\%=\frac{Md}{Mi}\times 100$$ 5

Where *S%* is the percentage solubility, *Md* is the mass of the dried filtrate and *Mi* is the initial mass of powder that was dissolved.

##### 2.1.4.6 HPLC Fingerprints of the RPE and of Spray-Dried Powders

The chromatographic conditions were as described by Jennyfer et al.,^27^ with some modifications. The analyses were performed using an HPLC Shimadzu Prominence LC-20A series coupled with an LC-6A double pump (Shimadzu Corporation, Kyoto, Japan). Separation was carried out on a C-18 column (Shimadzu Shim-Pack CLC (M) 4.6 mm × 25 cm, 5 μm, 100 Å) at 45 °C. The mobile phase consisted of a gradient system using water with 0.1% formic acid (A) and acetonitrile (B), with the following elution profile: 0–10 min with 20% B; 10–40 min, with linear increase to 50% B; 41–50 min with linear increase from 50 to 80% B; 51–60 min with linear increase to 100% B; 61- 62 min maintained at 100% B; 62–70 min with linear decrease to 20% B. Chromatograms were monitored at different wavelengths, and 281 nm was selected for analysis because it provided the best resolution of the main markers. Samples were prepared by dissolving 1 mL of the extract or 6 mg of spray-dried powder (based on the red propolis extract concentration) in a 5 mL volumetric flask, making up the volume with methanol. Subsequently, 230 μL of the stock solution was transferred to another 5 mL volumetric flask and diluted to volume with methanol. The final solution was filtered through a 0.45 μm membrane filter, and 20 μL was injected into the chromatograph for quantification. The presence of two key marker compounds, Liquiritigenin and Formononetin, was evaluated by preparing a 500 μg/mL solution of the respective standards. The retention times were recorded, and 500 μL of this standard solution was spiked into the red propolis extract during sample preparation. The resulting chromatograms were compared, and the peaks were validated accordingly. The chromatographic fingerprints of the spray-dried powders were then compared with those of the red propolis extract to assess chemical integrity and retention of bioactive compounds.

#### 2.1.5 Statistical Analysis

The statistical analysis of the experimental results was performed using a two-way analysis of variance (ANOVA) followed by a Bonferroni post hoc test. A significance level of 5% (p*<* 0.05) was adopted.

## 3 Results and Discussion

### 3.1 Total Flavonoid Content (TFC) of Red Propolis Extract

Flavonoids are the most common and widely distributed group of phenolics present in red propolis. They are among the most active compounds in red propolis and are responsible for several of its pharmacological activities. Flavonoids are marker compounds for red propolis, and their quantification is essential for characterizing the extract.^5^ The total flavonoid content (TFC) in plants is often determined colorimetrically after extraction. One of the most widely used methods for determining TFC in plant extracts is the aluminum chloride colorimetric assay, where Al (III) is utilized as a complexing agent.^28^

The total flavonoid content (TFC) of the red propolis extract (RPE) was determined from a calibration curve prepared with quercetin as the standard. From this calibration curve (R^2^ = 0.99451), the total flavonoid content was calculated using the equation of the curve and expressed as the quercetin equivalent (QE). The results indicated that RPE contains 13.11 mg/g QE. Similarly, Hernández et al.^29^ reported a range of 13 to 379 mg/g QE of flavonoid content in propolis collected nationwide, noting that flavonoid levels can be influenced by the type of vegetation from which bees collect. Elbaz et al.^30^ also reported a TFC of 13.19 mg/g CE (Catechin equivalent). In contrast, Barreto et al.^31^ found TFCs ranging from 77 to 104 mg/g QE in ethanolic (80%) extracts of red propolis samples exposed to ultrasonic technology. Woźniak et al.^32^ documented TFCs in propolis from different regions, ranging between 29.63 and 106.07 mg QE/g ethanol extract, attributing this variation to geographical origin. Furthermore, Bueno-Silva et al.^33^ reported seasonal variations in the chemical composition of Brazilian red propolis, suggesting that the collection season influences the extract’s composition.

### 3.2 Encapsulation of RPE in Gelucire

The encapsulation process was carried out in three stages, with samples collected at each stage for analysis. Stage 1 involved the formation of nanoparticles by dispersion and high-speed mixing on a magnetic stirrer, yielding the RPEG1 formulation. Stage 2 consisted of mixing with the Ultraturrax to produce the RPEG2 formulation. The final stage involved sonication, resulting in the RPEG3 formulation. The results are presented in Table 2.

**Table 2:** Characterisation of RPEG Formulations

	Average size (nm)	Zeta potential	PDI
RPEG1	644.1 ± 37.25	–32.87 ± 4.57	0.47 ± 0.04
RPEG2	612.9 ± 30.33	–37.37 ± 1.72	0.44 ± 0.03
RPEG3	459.7 ± 13.21	–36.93 ± 3.12	0.37 ± 0.04

The average sizes were 644.1 ± 37.25 nm for RPEG1, 612.9 ± 30.33 nm for RPEG2, and 459.7 ± 13.21 nm for RPEG3 (Table 3). According to Cao et al.,^34^ nanoparticles within the range of 100 to 500 nm can be absorbed in the epithelial walls of the intestine. While many effective oral delivery systems exceed 500 nm, improvements in bioavailability are generally attributed to enhanced dissolution and prolonged gastrointestinal residence rather than direct epithelial uptake.^35,36^ The nanoparticles produced in this study fall within the acceptable size range for oral administration.

**Table 3:** Particle Size Distribution of Powdered Samples

Powder sample	D_10_ (μm)	D_50_ (μm)	D_90_ (μm)	SPAN
SRPE1:1	1.81 ± 0.52	4.45 ± 0.40	9.62 ± 1.53	1.75 ± 0.07
SRPE1:2	1.4 ± 0.00	4.13 ± 0.00	9.12 ± 0.71	1.87 ± 0.17
SRPEG1:1	1.27 ± 0.24	3.74 ± 0.62	12.32 ± 0.46	2.99 ± 0.43
SRPEG1:2	1.92 ± 0.68	5.58 ± 1.56	13.84 ± 1.26	2.21 ± 0.52
GA/MDX/RPE1:2	1.07 ± 0.00	3.77 ± 0.40	10.64 ± 1.37	2.53 ± 0.10
MDX/RPE1:3	2.19 ± 0.00	4.49 ± 0.62	9.11 ± 1.16	1.54 ± 0.05
GARPE1:1	2.32 ± 0.00	5.46 ± 0.40	12.16 ± 0.95	1.80 ± 0.04
GARPE1:2	1.11 ± 0.00	4.23 ± 0.18	10.28 ± 0.00	2.17 ± 0.09
GARPEG1:1	2.24 ± 0.00	7.01 ± 0.15	15.29 ± 0.06	1.86 ± 0.05
GARPEG1:2	1.81 ± 0.91	5.87 ± 1.58	13.97 ± 1.26	2.14 ± 0.52

There was no statistical difference in the sizes of RPEG1 and RPEG2, suggesting that Stage 2 could be excluded to enhance the process’s scalability. Fewer processing steps make the method more applicable on an industrial scale. Additionally, many bubbles were produced during Stage 2, which could negatively impact the sonication process. Therefore, high-speed mixing with the Ultraturrax in Stage 2 may be unnecessary, as the results suggest this step is not required.

The low polydispersity index (PDI) of 0.37 for the final formulation indicates that it is homogeneous.^37^ Furthermore, the negative zeta potential (−36.93 ± 3.12) is sufficiently high, indicating that the formulation is stable with a low probability of aggregation over a short period of time.^38^ Similar results were reported in the study done by Fatemah et al.,^39^ which involved the formation of nanoparticles with red propolis.

### 3.3 Microencapsulation of RPE

Ten different powdered samples were obtained by varying the ratios of carriers (arabic gum, OSA-modified starch, and maltodextrin) during spray drying of Gelucire-based formulations. Formulations containing the carriers, but without Gelucire were used as controls for comparison. It is important to note that spray-drying pure RPE (without carriers) was not feasible due to the extract’s resinous nature and high adhesiveness, which prevent the formation of a free-flowing powder.

#### 3.3.1 Yield after Spray-Drying

Carrier agents such as maltodextrin and gum arabic improve spray-drying yield by reducing stickiness and product loss, thereby enabling more efficient recovery of dried powders^40^ (Figure 1). This effect is largely attributed to their ability to increase the glass transition temperature of the feed, preventing the propolis extract from transitioning into a sticky, rubbery state during drying and adhering to the chamber walls. Maltodextrin has been reported to enhance the yield of spray-dried powders,^41^ consistent with the results observed in this study. Additionally, Yousefi et al.^42^ reported that increasing carrier concentration is directly proportional to yield, which explains the superior performance of maltodextrin among the three carriers, as it was used at the highest concentration (Table 1). The lowest yield was obtained for the formulation containing modified starch (octenyl succinic acid anhydrous starch), which aligns with the findings of Du Jing et al.,^43^ who reported substantially lower yields for starch-based carriers compared with maltodextrin and gum arabic. Furthermore, powders obtained from RPEG formulations generally exhibited higher yields than those from the corresponding formulations without Gelucire, suggesting that the presence of Gelucire enhances yield during spray drying. This is likely due to its emulsifying properties, which promote better entrapment of sticky lipophilic components within the carrier matrix and further reduce wall deposition.

**Figure 1:**
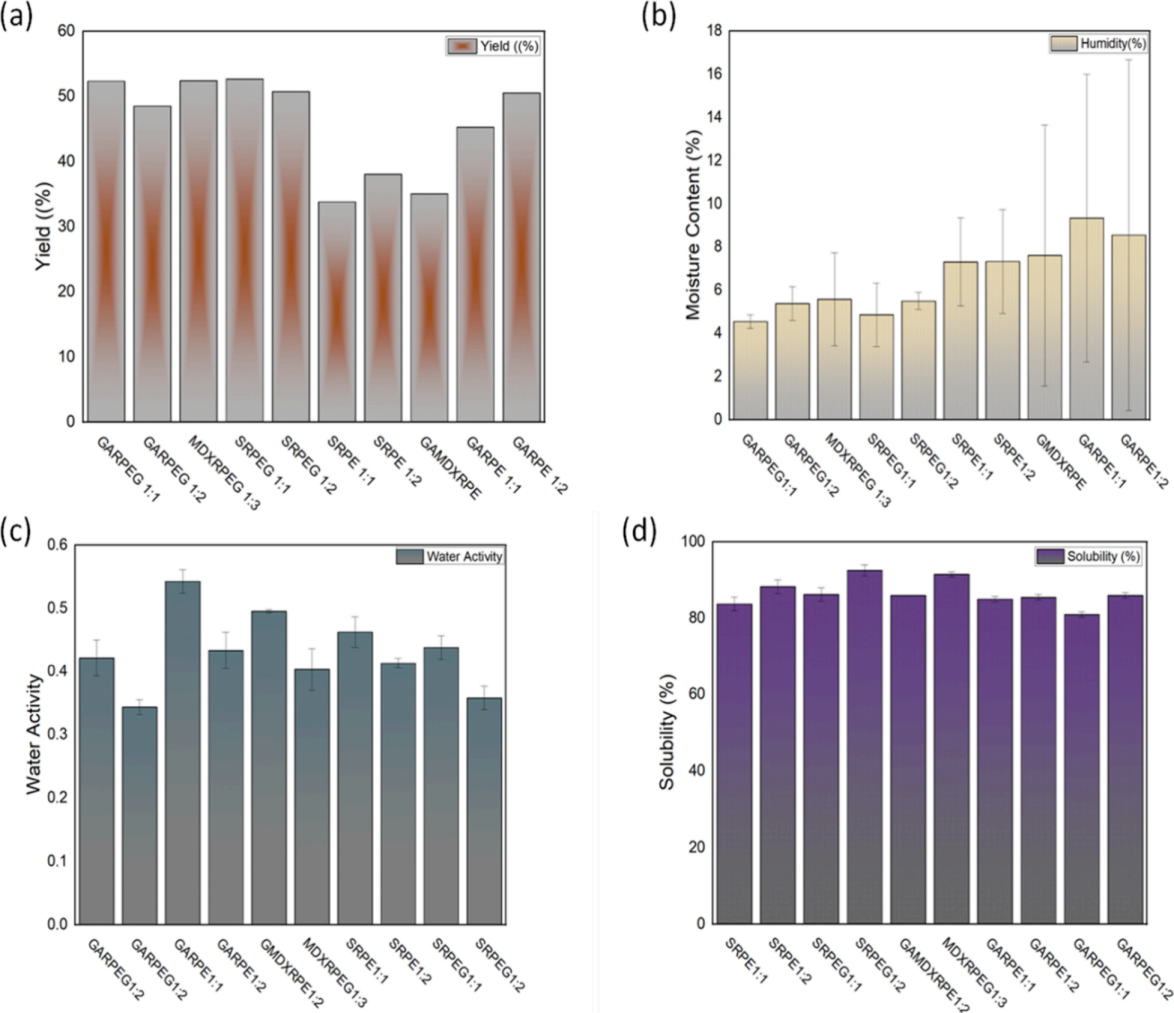
(a) Yield after spray-drying; (b) moisture content; (c) water activity; (d) aqueous solubility of spray-dried powders.

#### 3.3.2 Moisture Content

All powders derived from the RPEG formulations, regardless of the carrier used, exhibited significantly lower (*p* < 0.05) moisture content than plain red propolis powder (Figure 1b). These results indicate that the inclusion of Gelucire in the formulation reduces moisture content. Recent spray-drying studies commonly report residual moisture levels of 3 to 5% (w/w) as appropriate for ensuring powder stability during storage, particularly for botanical and nutraceutical formulations.^44,45^ In this context, the GARPEG1:1 and SRPEG1:1 powder samples fell within this optimal range, suggesting that these formulations are the most stable and least prone to deterioration during storage. While research by Tonon et al., Mohammad et al., and Cordin et al.^46–48^ indicates that spray-drying parameters (such as inlet temperature) significantly affect moisture content, the carrier type and ratio played a key role in this study. Specifically, lower carrier ratios yielded lower humidity readings. Consistent with Arya et al.,^49^ higher concentrations of carrier agents lead to increased moisture content in final powders due to the carriers’ moisture retention capabilities. The lowest moisture content was observed in GARPEG 1:1 (4.55 ± 0.31% w/w) while the highest was in GARPE1:1 (9.34 ± 6.66% w/w). Compared with modified starch and maltodextrin, arabic gum is more hygroscopic, producing powders with higher residual moisture. This behavior can be attributed to its highly branched molecular structure and the abundance of hydrophilic groups that bind or absorb ambient water molecules.^50^ The reduced residual moisture of the Gelucire-based powders can be attributed to the excipient’s amphiphilic nature and its ability to form a cohesive lipid–PEG matrix that limits water retention during drying. Such reduction is advantageous, as moisture levels below 5% (w/w) enhance powder stability, prevent caking, and improve handling and shelf life. These qualities are essential for both pharmaceutical and nutraceutical applications. These findings align with previous reports that Gelucire matrices stabilize amorphous systems and improve the technological performance of spray-dried bioactive powders.^7,12,13^

#### 3.3.3 Water Activity

It strongly influences microbial growth and chemical stability, and it can also affect physical properties such as flowability and solid-state stability.^51^ Water activity (aw) reflects the amount of free or available water in a pharmaceutical or food product. In this study, water activity was assessed specifically to evaluate the microbial and storage stability of the spray-dried powders, considering them as intermediates or final products. The a_w_ of all the powders generally fell within the acceptable range for pharmaceutical powders (Figure 1c). According to Sandle,^52^ powders with water activity above 0.7 can support microbial growth. It is, however, noteworthy that all the powdered formulations containing Gelucire exhibited lower water activity (0.34–0.44) compared to those without Gelucire, indicating that Gelucire-based formulations offer superior chemical and microbial stability. The powder with the highest water activity (a_m_ = 0.542) was GARPE1:1, likely due to the effect of arabic gum as the carrier. The water activity of arabic gum has been reported to range from 0.52 to 0.56.^53^ A significant difference (*p* < 0.05) was observed when comparing this sample (GARPE1:1) with its Gelucire-based counterpart (GARPEG1:1), demonstrating that Gelucire improves the quality of the powdered formulation. Furthermore, since the red propolis is highly hydrophobic,^54^ most of the water present in the formulations exists in free form. Hence, the inclusion of Gelucire 50/13 appears to promote the formation of a more structured matrix, limiting moisture mobility and contributing to improved stability and reduced water activity.

#### 3.3.4 Aqueous Solubility of Powders

Spray-drying of hydrophobic extracts, such as RPE, typically yields amorphous powders with high surface area and improved wettability.

These physical characteristics significantly enhance the dissolution rate and apparent solubility of the bioactive compounds in aqueous media. The choice of carrier materials significantly influences powder solubility, often leading to considerable variation.^49,55^ In this study, all the spray-dried powder samples exhibited water solubility at 30 °C, ranging from 81% to 92.5% (Figure 1d). The highest solubility was observed in SRPEG 1:2, followed by MDXRPEG 1:3. In contrast, the lowest solubility was recorded in GARPEG 1:1. This suggests that powder is mainly dependent on the properties of the carriers, with arabic gum exhibiting significantly lower solubility (*p* < 0.05) compared to maltodextrin and modified starch. This finding is consistent with Milton et al.^26^ who reported that while spray-dried mango juice powders formulated with gum arabic showed good solubility, maltodextrin exhibited superior solubility. Similarly, Tran et al.^56^ observed improved solubility in spray-dried lemongrass extract powders when maltodextrin was used as a carrier instead of gum arabic. Srinivas et al.^57^ also reported an increase in water solubility with higher maltodextrin concentrations. Furthermore, Mohammad et al.^55^ indicated that starch-based carriers exhibit high solubility due to their physical structure. Additionally, OSA modification introduces hydrophilic groups into starch molecules, enhancing their amphiphilic nature, stability, and solubility.^58^ Gelucire 50/13 contributed to aqueous solubilization and matrix formation across all the RPEG formulations, facilitating molecular dispersion of the bioactive compounds and underscoring their suitability for oral and functional food applications where both bioactive delivery and technological performance are critical.

### 3.4 Particle Size Distribution of Spray-Dried Powders

Figure 2 shows the typical optical photomicrographs of the spray-dried red propolis extract formulations obtained. The images were processed using Image-Pro Plus 4.5 software, which permitted the Determination of the size distribution of the powdered product and the corresponding values of D_10_, D_50_, D_90_, and SPAN (Table 3). The spray-dried powders had a mean diameter range of 1 to 20 μm. The uniformity in the range can be attributed to the same spray-drying conditions, as the particle size of powders obtained during spray drying is primarily influenced by process parameters, such as drying temperature, flow rate, pump speed, and atomization speed.^20,59,60^ The SPAN values of the powders ranged from 1.5 to 3, indicating a moderate to broad particle size distribution, with SRPEG 1:1 exhibiting the broadest distribution. The particle size distributions of powders generally influence their application, as they affect the flow properties, packing density, and can also influence significant properties of the final products.^61,62^

**Figure 2:**
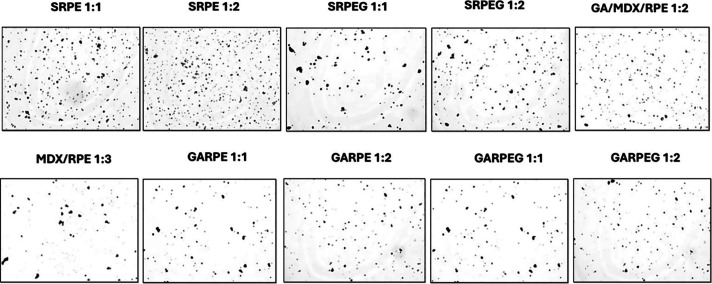
Typical photomicrographs of the spray-dried red propolis extract formulations obtained at a magnification of 50×.

### 3.5 Flow Properties of the Spray-Dried Powders

The bulk density of the powders ranged from 0.17 to 0.27 g/mL, and the tapped density ranged from 0.21 to 0.31 g/mL. It has been observed that spray-dried powders generally have lower bulk densities.^63,64^ The bulk and tapped densities of RPE powders with modified starch and maltodextrin carriers were significantly lower (*p* < 0.05) than those with gum arabic as the carrier. According to Srinivas et al.,^57^ the use of maltodextrin as a carrier results in powders with lower bulk density. Vishnuvardhan^65^ also reported that the bulk density of sweet orange powder decreased with increasing maltodextrin concentration, probably due to a reduced tendency for particles to stick together, thereby increasing particle volume due to entrapped air. This trend is consistent across bulk and tapped densities for powders containing maltodextrin and arabic gum. Furthermore, the presence of Gelucire had a significant impact, particularly when comparing powders with and without Gelucire using arabic gum as the carrier. This difference was not observed with powders produced using maltodextrin and modified starch, but was clearly evident in powders with gum arabic as the carrier. GARPEG powders had significantly lower bulk and tapped densities compared to GARPE and GAMDXRPE powders. GARPEG powders had larger particles than GARPE powders, highlighting the effect of particle size on powder density.^66^ Largely low bulk density of the powders indicates their structural strength and resistance to collapse in containers. Low bulk densities also influence the flowability of the powders.^67,68^

The Hausner ratio and the Carr index were derived from the tapped and bulk densities (Table 4), indicating the flow properties and compressibility of the powders. The results demonstrated that the powders exhibit acceptable flow properties, suggesting their potential usefulness in industrial and pharmaceutical processes.^20,58,69,70^ In a free-flowing powder, the Carr Index (CI) value would be smaller, as the bulk density and tapped density of the powder would be closer in value. Conversely, in a poorly flowing powder, the difference between the bulk and tapped densities would be more pronounced, as greater interparticle interactions cause larger Carr index values. Furthermore, the moisture content of the powder significantly affects flowability, sticking, and caking properties. The higher the moisture content, the greater the cohesive forces, resulting in poor flowability.^65^

**Table 4:** Bulk and Tapped Density of Spray-Dried Powders (g/mL), Hausner’s Ratio, and Carr’s Index

Samples	Bulk density (g/cm^3^)	Tapped density (g/cm^3^)	Hausner’s ratio (−)	Carr’s index (−)
SRPE1:1	0.176	0.216	1.23	18.6
SRPE1:2	0.181	0.228	1.26	20.48
SRPEG1:1	0.184	0.225	1.22	18.29
SRPEG1:2	0.177	0.212	1.2	16.47
GA/MDX/RPE1:2	0.244	0.28	1.15	12.9
MDXRPE1:3	0.197	0.231	1.17	14.47
GARPE1:1	0.255	0.289	1.13	11.86
GARPE1:2	0.265	0.309	1.16	14.04
GARPEG1:1	0.169	0.206	1.22	17.98
GARPEG1:2	0.179	0.211	1.18	15.48

### 3.6 HPLC Fingerprints of the RPE and of Spray-Dried Powders

Encapsulation involves multiple processes aimed at enhancing the pharmacological efficacy of the extract by addressing its limitations. Therefore, it is imperative to confirm that the chemical integrity of the encapsulated extract is maintained postencapsulation. A qualitative HPLC analysis was performed to compare the chemical profiles of the extract and the spray-dried powders, with compound identities confirmed by their UV spectra. Figure 3 presents the HPLC chromatographic profiles of RPE, GARPE, GARPEG, SRPE, and SRPEG samples, acquired at 281 nm. The profiles were compared with the chemical profile of Brazilian red propolis reported by Jennyfer et al.^25^ Peaks corresponding to liquiritigenin and formononetin were identified using reference standards, with similar retention times (Liquiritigenin between 12.5 and 13 min and Formononetin at near 24. 24.5 min), peak shapes, and areas observed across all samples. Minor differences in peak intensity and retention time may be attributed to differences in wall materials and the presence of Gelucire during spray drying.^71^ The spiking of the extract with standard compounds also helped to validate the peaks identified in the chromatogram to Formononetin and Liquiritigenin. The UV spectra of the identified peaks in the powders closely matched those of the formononetin (isoflavone) and liquiritigenin (flavonoid) standards, confirming the retention of these bioactive compounds. This spectral consistency, illustrated in Figure 3, demonstrates that the chemical profile of the extract and all encapsulated powders remained unchanged, indicating that the encapsulation process preserved the integrity of the pharmacologically active markers.

**Figure 3:**
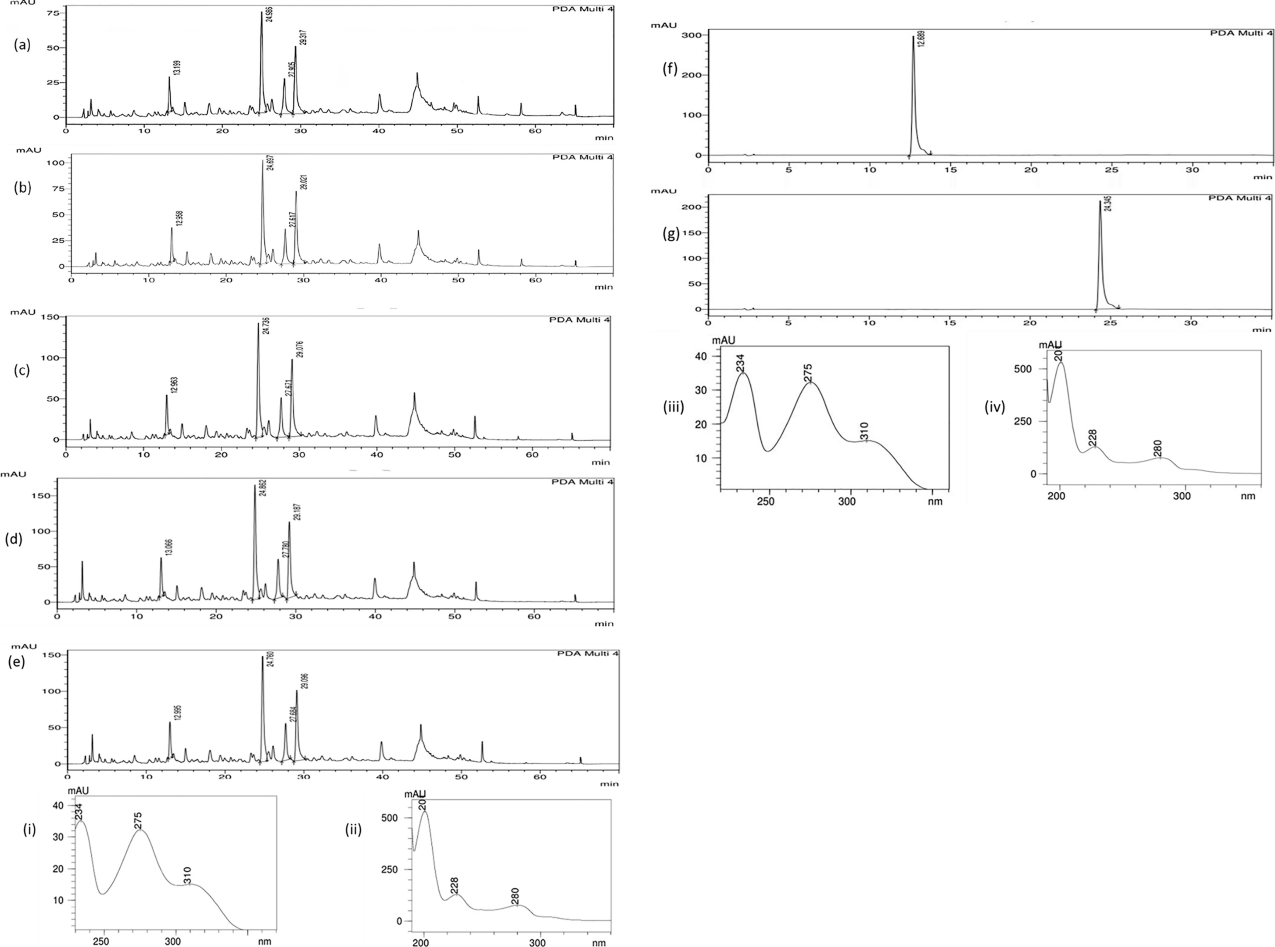
Chemical profile of the extract and spray-dried formulations. (a) RPE, (b) GARPE, (c) GARPEG, (d) SRPE, (e) SRPEG, (f) liquiritigenin standard, and (g) formononetin standard. (i, ii) UV spectra of liquiritigenin and formononetin in the extract; (iii, iv) UV spectra from the reference compounds.

## 4 Conclusions

The results of this study indicate that encapsulating red propolis extract (RPE) with Gelucire 50/13 produced stable formulations. Upon spray-drying, these formulations exhibit optimized physicochemical properties compared to plain RPE encapsulations. Additionally, the qualitative analysis suggests that the chemical integrity of RPE was not compromised during the encapsulation process. Consequently, Gelucire 50/13 has been identified as a viable and effective encapsulating agent for red propolis.
